# The Influence of Breed, Sex, Origin and Housing Conditions on Undesirable Behaviors in Ancient Dog Breeds

**DOI:** 10.3390/ani11051435

**Published:** 2021-05-17

**Authors:** Anna Wójcik, Kinga Powierża

**Affiliations:** Department of Animal and Environmental Hygiene, Faculty of Animal Bioengineering, University of Warmia and Mazury in Olsztyn, 5 Oczapowski Street, 10-719 Olsztyn, Poland; kinga.powierzaaa@gmail.com

**Keywords:** ancient dogs, undesirable behaviors, breed, sex, origin, housing conditions

## Abstract

**Simple Summary:**

Abnormal, repetitive behaviors often pose a problem for dog owners. Such behaviors are considered undesirable if they pose a nuisance or a danger to humans. This study aimed to identify factors (breed, sex, origin, housing conditions) and situations that contribute to undesirable behaviors, such as aggression towards humans and other dogs/animals, separation anxiety, excessive vocalization, and oral and locomotion behaviors in the ancient dog breeds Akita, Alaskan Malamute, Basenji, Samoyed and Siberian husky. Undesirable behaviors were analyzed based on the results of a survey involving dog owners. Undesirable behaviors were most frequently reported in Akitas, Siberian huskies and Samoyeds, and they were more prevalent in males than in females and dogs living indoors with or without access to a backyard. Aggressive behaviors towards other dogs and animals, excessive vocalization and undesirable motor activities posed the greatest problems in ancient dog breeds.

**Abstract:**

Abnormal repetitive behaviors often pose problems for dog owners. Such behaviors are considered undesirable if they pose a nuisance or a danger to humans. Ancient dog breeds are intelligent, sociable, active, boisterous and need regular outdoor exercise, but are also independent and reluctant to follow commands. This study aimed to identify factors (breed, sex, origin, housing conditions) and situations that contribute to undesirable behaviors, such as aggression towards humans and other dogs/animals, separation anxiety, excessive vocalization, and oral and locomotion behaviors in Akita, Alaskan Malamute, Basenji, Samoyed and Siberian husky. Undesirable behaviors in dogs were analyzed based on the results of 897 questionnaires. Breed influenced aggressive behavior towards other dogs/animals, aggression towards humans, undesirable oral and locomotion behaviors, and excessive vocalization. Aggressive behaviors were more prevalent in females than in males. Housing conditions were linked with aggression towards other dogs/animals, aggression at mealtime, and excessive vocalization. Undesirable behaviors were most frequently reported in Akitas, Siberian huskies and Samoyeds, and they were more prevalent in males than in females and dogs living indoors with or without access to a backyard. Aggressive behaviors towards other dogs and animals, excessive vocalization and undesirable motor activities posed the greatest problems in ancient dog breeds.

## 1. Introduction

Abnormal repetitive behaviors often pose a problem for dog owners [1]. Such behaviors are considered problematic if they pose a nuisance or a danger to household members [2]. In turn, behaviors that are unacceptable for dog owners are described as undesirable, even if they are consistent with an animal’s ethogram of normal behavior [3,4,5]. Examples of undesirable behavior include excessive activity, excitation, vocalization or unprovoked aggression [6,7]. Stereotypies are repetitive, constant acts that serve no obvious purpose [8]. In turn, compulsive behaviors are defined as repetitive acts that exceed normal functions and disrupt daily activities [9,10,11]. Compulsive behaviors can be both physical and psychological. They are observed in many animal species, including companion animals, zoo animals and livestock [10,12]. According to Luescher et al. [13], stereotypic behaviors may be present in 1.19% to 3.3% of domestic animals in the USA. According to many authors, behavioral problems emerge in stressful situations when an animal cannot physically escape from the source of stress or in the absence of exciting stimuli [10,14,15,16]. Such problems also arise when animals are unable to cope with sudden changes in their environment. Withheld rewards also cause frustration, which can lead to stereotypes.

Canine behavioral problems are affected by various factors, such as breed, age, sex, health condition, housing conditions, and the relationship between a dog and its owner [10,17,18,19]. Dogs that are not exposed to various environmental stimuli during socialization in early life are more likely to develop behavioral problems in adulthood [4,15]. Such behaviors include over-grooming (excessive licking), excessive motor behavior (tail chasing, spinning in circles), hunting, feeding (guarding food and the food bowl, pica) and sexual behavior. However, aggression and excessive vocalization are the most frequent problematic and undesirable behaviors in dogs [15,16,20,21]. Owners do not always recognize changes in a dog’s behavior or interpret such changes as harmless play [22,23]. In addition, most owners make attempts to eliminate undesirable behaviors only when their symptoms are intensified and become predominant in a dog’s daily behavior [24]. Dog owners claim that abnormal behavior interferes with a dog’s daily functioning by preventing the animal from eating, playing and interacting with people [11,25], and dogs displaying behavioral problems are often placed in a shelter or euthanized [4,21,26,27]. However, some owners seek the advice of canine behaviorists. In addition to the home environment, compulsive and stereotypic behaviors are often observed in shelters, where dogs live in small, confined spaces with other animals in an unstimulating environment [28,29,30].

Ancient dog breeds are highly demanding when it comes to housing conditions and relationships with humans. These breeds are most closely related to wolves [31], which is reflected in their character traits. Parker et al. [31] studied 85 dog breeds and identified 14 ancient breeds, including Akita, Alaskan Malamute, Basenji, Samoyed and Siberian husky. These breeds are intelligent, sociable, active, boisterous and need regular outdoor exercise. Ancient dog breeds are independent and reluctant to follow commands. For these reasons, ancient dog breeds may be difficult to train. In the work of Coile [32], ancient dog breeds scored one to three points on a five-point scale for ease of training. This result indicates that ancient breeds are difficult or very difficult to train. Ancient breeds are also highly territorial and have a strong hunting instinct. These character traits can lead to aggressive behavior towards humans and other dogs and animals [32]. Many dog buyers are not aware of the characteristics and specific needs of ancient breeds. Dogs can display undesirable behaviors if their biological and behavioral needs are not met [33,34].

Because ancient dog breeds have specific character traits [32] and that behavioral problems in these breeds have never been discussed in the literature, this study was undertaken to explore undesirable behaviors in popular ancient dog breeds in Poland: Akita, Alaskan Malamute, Basenji, Samoyed and Siberian husky. According to the Polish Kennel Club, there are 2726 dogs of ancient breeds in Poland (including 1080 males and 1646 females). However, this figure accounts for the animals registered with the Polish Kennel Club, whereas the population of the remaining ancient breeds in Poland is difficult to estimate due to insufficient data.

This study aimed to identify factors (breed, sex, origin, housing conditions) and situations that contribute to undesirable behaviors, such as aggression towards humans and other dogs/animals, aggression at mealtime, separation anxiety, excessive vocalization, and undesirable oral and locomotion behaviors in ancient dog breeds: Akita, Alaskan Malamute, Basenji, Samoyed and Siberian husky.

## 2. Materials and Methods

### 2.1. Data Collection

Undesirable behaviors were analyzed in selected ancient dog breeds, including Akita, Alaskan Malamute, Basenji, Samoyed and Siberian husky, based on an online survey conducted among the users of social networking sites dedicated to ancient dog breeds. The survey was anonymous, and it was conducted between 18 August 2019 and 27 September 2019. A total of 897 respondents participated in the survey.

The questionnaire is presented in Supplementary Materials. The survey was composed of two parts. The first part focused on factors that could contribute to undesirable behaviors, including breed, sex, origin, previous and present housing conditions. The respondents could select one of the following options to describe the dog’s origin: kennel registered with the Polish Kennel Club, private owner, unregistered kennel, foundation, shelter, or origin unknown. The following options could be selected to describe the dog’s previous and present housing conditions: indoors without backyard access (apartment), indoors with backyard access (house), outdoors in a kennel, outdoors without confinement, outdoors with confinement, other. In the question addressing the animal’s housing conditions, the respondents were asked to describe how dogs were kept as puppies or the last known housing conditions.

In the second part of the survey, the respondents were asked to describe undesirable behaviors and indicate situations in which such behaviors occurred (Table 1).

The respondents could describe other behavioral problems by selecting the “other” option.

### 2.2. Statistical Analysis

The results of the questionnaire survey were analyzed using Pearson’s chi-squared test in the Statistica 13.3 program (TIBCO Software Inc.,Palo Alto, CA, USA) [35]. The relationships between the following variables: breed, sex, origin, previous and present housing conditions, vs. undesirable behaviors: aggression towards other dogs/animals, aggression towards humans, aggression at mealtime, separation anxiety, undesirable oral and locomotion behaviors, excessive vocalization (yes/no) were analyzed. The results were regarded as significant at *p* ≤ 0.05. The percentage of undesirable behaviors and the situations in which undesirable behaviors occurred were listed in the Excel spreadsheet program (MS Office 365). The percentage of undesirable behaviors in each breed, each category of undesirable behaviors and housing conditions was calculated relative to the total number of reported undesirable behaviors and relative to undesirable behaviors in each sex. The percentage of situations in which undesirable behaviors were reported and specific behaviors in each category was calculated relative to undesirable behaviors reported separately for each dog breed.

## 3. Results

The conducted survey revealed differences in undesirable behaviors in the analyzed ancient dog breeds. Breed influenced five of the six analyzed undesirable behaviors (Table 2). Aggressive behavior towards other dogs/animals was reported in 58.89% of Akitas, 46.55% of Alaskan Malamutes and 54.69% of Basenjis (χ^2^ = 122.739, *p* ≤ 0.001). Aggressive behavior towards other dogs and animals was least prevalent in Samoyeds and Siberian huskies (11.47% and 34.96%, respectively). Samoyeds were also least aggressive towards humans (5.5%) (χ^2^ = 12.997, *p* ≤ 0.011). Aggressive behavior towards humans was most frequently displayed by Alaskan Malamutes (17.24%) and Akitas (13.04%).

Undesirable behaviors in dogs include oral and locomotion behaviors as well as excessive vocalization. Breed exerted a significant effect on these behaviors. Stereotypic motor behaviors were most prevalent (χ^2^ = 10.018, *p* ≤ 0.040) in Samoyeds (29.36%) and Akitas (27.27%) and were least prevalent in Siberian huskies (17.89%). Aggression at mealtime (χ^2^ = 23.231, *p* ≤ 0.001) was less frequently reported than undesirable motor activities, excluding Alaskan Malamutes and Siberian huskies. These behavioral problems were noted in 29.31% of Alaskan Malamutes and 20.33% of Siberian huskies.

Excessive vocalization was most often observed in Samoyeds (44.04%), and it was least prevalent in Akitas (17.39%; χ^2^ = 44.699, *p* ≤ 0.001).

The incidence of separation anxiety was similar in the analyzed breeds, ranging from 9.88% in Akitas to 17.89% in Samoyeds.

An analysis of the relationship between sex and undesirable behaviors (Table 3) revealed that sex significantly contributed (χ^2^ = 4.821, *p* ≤ 0.028) to aggression towards other dogs and animals. Aggressive behaviors were more prevalent in females (64.76%) than in male dogs (57.51%).

The incidence of the remaining undesirable behaviors was similar in both sexes.

An analysis examining the extent to which a dog’s origin contributed to undesirable behaviors did not reveal differences between groups (Table 4). Previous housing conditions were not linked with behavioral problems (Table 5).

An analysis examining the influence of present housing conditions on undesirable behaviors (Table 6) confirmed that this factor contributed to aggressive behavior towards other dogs and animals (χ^2^ = 15.236, *p* ≤ 0.002), aggression at mealtime (χ^2^ = 8.509, *p* ≤ 0.037) and excessive vocalization (χ^2^ = 9.601, *p* ≤ 0.022). Aggressive behavior was most prevalent in dogs living outdoors without confinement (53.85%) and dogs living indoors with backyard access (43.36%). This behavioral problem was reported in 33.89% of dogs living indoors without backyard access. Dogs living outdoors in kennels were the least aggressive (14.29%). Aggression at mealtime was most prevalent in dogs living indoors with backyard access (21.33%) and dogs living outdoors (17.95%). This problem was least frequently noted in dogs living indoors without backyard access (13.74%).

An analysis of the relationships between housing conditions and excessive vocalization produced the opposite results. Dogs kept in outdoor kennels (35.71%), and dogs living indoors without backyard access (31.71%) tended to be most vocal. Excessive vocalization was less prevalent in dogs living in a more stimulating environment, including dogs kept outdoors and dogs living indoors with access to a backyard (22.75% and 25.64%, respectively).

Undesirable behaviors in ancient dog breeds were also examined in a situational context. The respondents reported a total of 1939 undesirable behaviors (1062 in males and 877 in females). Many dogs displayed multiple behavioral problems.

**Figure 1:**
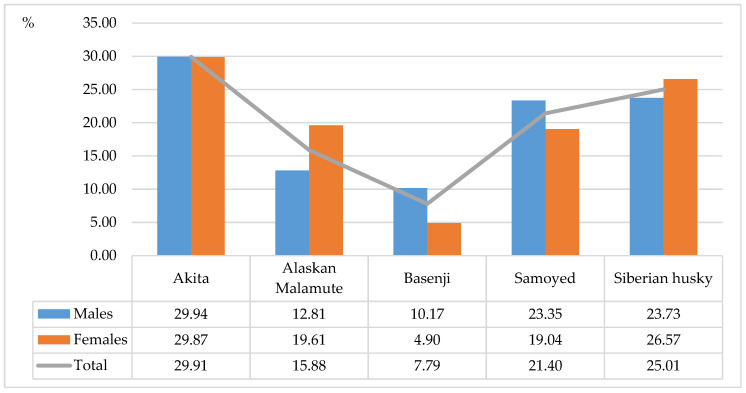
Incidence of undesirable behaviors in the examined dog breeds (%).

**Figure 2:**
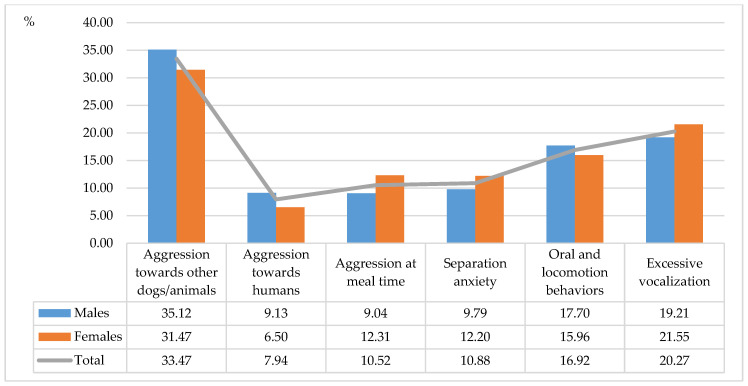
Incidence of undesirable behaviors in each category of behavioral problems (%).

**Figure 3:**
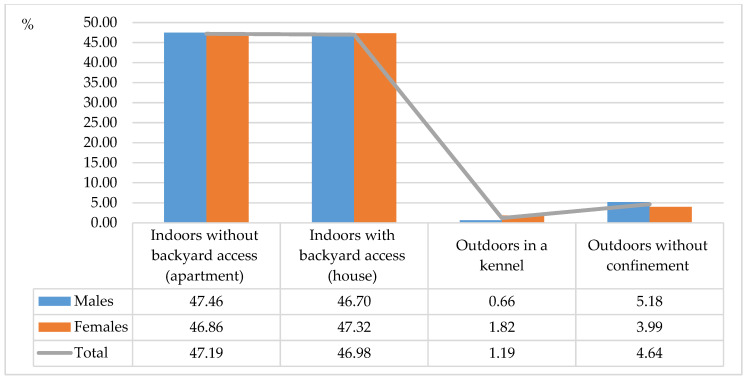
Incidence of undesirable behaviors in dogs living in different housing conditions (%).

The highest number of undesirable behaviors was reported in Akitas (29.91%), Siberian huskies (25.01%) and Samoyeds (21.4%) (Figure 1). The smallest number of undesirable behaviors was observed in Basenjis (7.79%) and Alaskan Malamutes (15.88%). The most prevalent behavioral problems were aggression towards other dogs/animals (33.47%), excessive vocalization (20.27%) and undesirable oral and locomotion behaviors (16.92%) (Figure 2). An analysis examining the impact of housing conditions on behavioral problems in ancient dog breeds revealed that undesirable behaviors were most common in dogs living indoors without backyard access (47.19%) and dogs living indoors with backyard access (46.98%) (Figure 3), which could be attributed to limited living space and low levels of physical activity.

**Figure 4:**
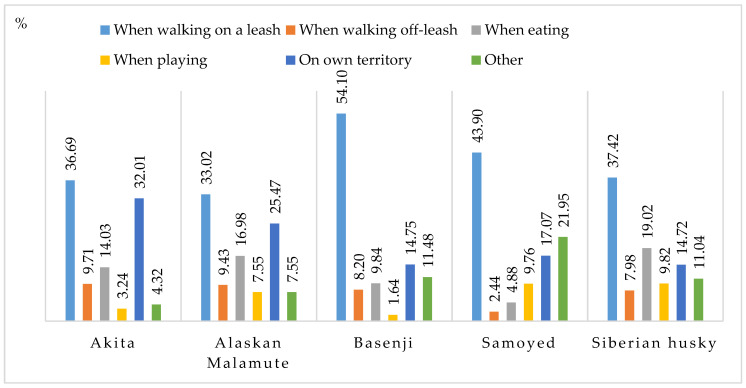
Situations that elicit an aggressive response towards other dogs/animals (%).

The incidence of aggressive behavior towards other dogs and animals was analyzed when dogs were walked on a leash, walked off-leash when eating, playing, on their territory, and in other situations (Figure 4). In more than 30% of the examined cases, aggressive behavior was displayed when the dog was walked on a leash. The above scenario accounted for more than 50% of undesirable behaviors in Basenjis. The incidence of aggressive behavior ranged from 2.44% (Samoyeds) to 9.71% (Akitas) when dogs were walked off-leash. Aggressive behaviors towards other animals were also common on a dog’s territory, and they accounted for more than 30% of the cases in Akitas and more than 25% of the cases in Alaskan Malamutes. When asked about other forms of aggression towards other dogs and animals, the respondents claimed that their dogs were aggressive only in response to aggressive behavior displayed by other dogs. In some cases, dogs were aggressive to strangers who approached the owner (for example, during a walk), which was perceived probably as a potential threat by the dog.

**Figure 5:**
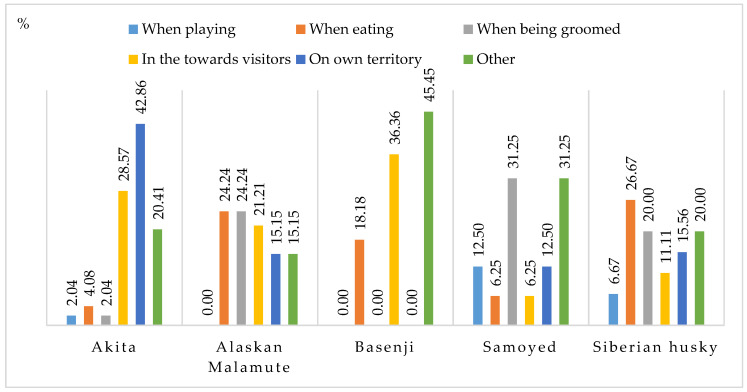
Situations that elicit an aggressive response towards humans (%).

Aggressive behavior towards humans was analyzed during playing, eating, grooming, guest visits, on own territory and in other circumstances (Figure 5). In Akitas, aggression towards visitors and aggression on their territory accounted for more than 70% of all aggressive behaviors towards humans in this breed. Aggression towards humans on the dog’s territory accounted for 42.86% of the cases, and aggression towards visitors accounted for 28.57% of the cases in Akitas. Aggressive behaviors towards visitors were also frequent in Basenjis (36.36%) and Alaskan Malamutes (21.21%). The respondents who were asked about other reasons for aggression towards humans pointed out that ancient dog breeds are aggressive not only towards strangers but also towards people they know. An analysis of aggressive behaviors during grooming indicates that ancient dog breeds, particularly Alaskan Malamutes, Samoyeds and Siberian huskies, experience discomfort while being groomed (24.24%, 31.25% and 20%, respectively).

**Figure 6:**
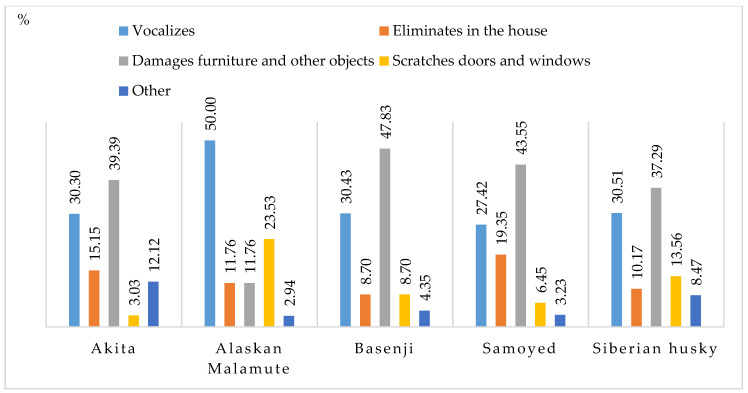
Undesirable behaviors when dogs are left alone at home (indicative of separation anxiety) (%).

Problematic behaviors that could indicate separation anxiety included vocalization, elimination in the house, damaging furniture and other objects, scratching doors and windows, and other (Figure 6). The most prevalent behavioral problems in this category were vocalization (from 27.42% in Siberian huskies to 50% in Alaskan Malamutes) and damaging furniture and other objects (from 11.76% in Alaskan Malamutes to 47.73% in Basenjis). According to the respondents, other behaviors indicative of separation anxiety were observed in early life. These problems disappeared after training and when dogs became accustomed to being left alone at home or in the backyard.

**Figure 7:**
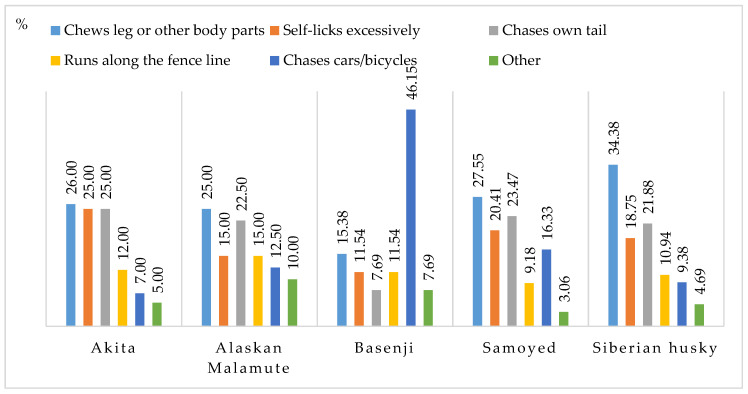
Oral and locomotion behaviors (%).

Undesirable motor activities included self-chewing, excessive licking, tail chasing, running along the fence line, chasing cars, and other (Figure 7). The first three behaviors were most often reported in the survey. They were least prevalent in Basenjis, where car and bicycle chasing was most frequently reported (46.15% of the cases). Other undesirable behaviors included hunting, spinning or chewing on the leash during walks.

**Figure 8:**
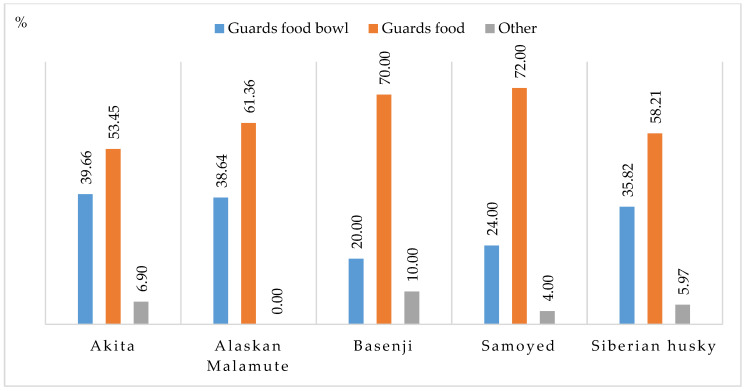
Aggression at meal time (%).

Aggression at mealtime included food or food bowl guarding and other undesirable behaviors (Figure 8). The reported cases of food guarding ranged from 53.45% in Akitas to 72% in Samoyeds. Other undesirable feeding behaviors included eating every other day or eating at night. Such behaviors could be caused by health problems, and in such cases, the owner should consult a veterinary practitioner.

**Figure 9:**
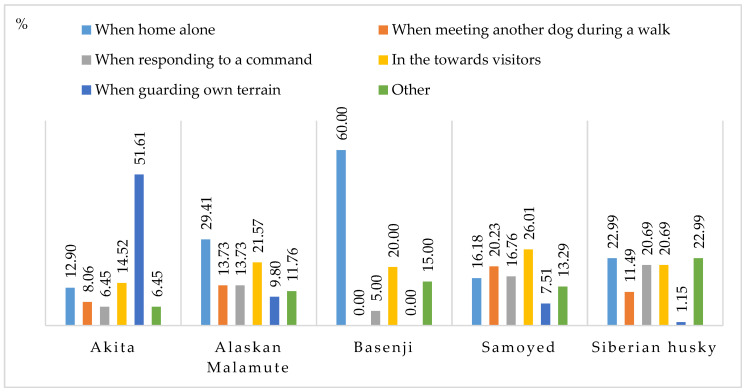
Situations that elicit excessive vocalization (%).

Excessive vocalization is a frequent problem in dogs. In this study, Samoyeds were the most vocal breed (Table 2). Samoyeds were excessively vocal towards visitors (26.01%) and when meeting other dogs during walks (20.23%) (Figure 9). In 60% of the cases, Basenjis vocalized excessively when left at home alone. Excessive vocalization and behaviors that indicate separation anxiety suggest that Basenjis could be more susceptible to stress resulting from loneliness (Figure 6). In turn, Akitas were most vocal when guarding their territory (51.61%). In this breed, territorial aggression was also linked with aggression towards other dogs/animals (Figure 4) and humans (Figure 5). Other situations that provoked excessive vocalizing included overexcitement during walks, excitement when the owner returned home and attempted to elicit play or a treat from the owner. Many respondents believed that ancient dog breeds vocalize without an obvious reason.

## 4. Discussion

Aggressive behavior towards other dogs, animals and humans are one of the most common behavioral problems in dogs [4,20,36,37,38]. An Australian study demonstrated that aggression (37%) and excessive vocalization (14%) were the most prevalent behavioral problems in dogs of various breeds living in the area of Brisbane and Gold Coast [4]. Ancient dog breeds (Alaskan Malamutes and Siberian husky) accounted for only 0.43% and 1.40% of the studied population, respectively, but they displayed the same behavioral problems as dogs of more popular breeds.

Our results revealed certain differences in aggressive behaviors towards other dogs/animals in the studied ancient dog breeds. Aggressive behaviors were reported in 50% of Akitas, Alaskan Malamutes and Basenjis, a high and worrying number. Aggressive behavior can be attributed to the specific character traits of ancient dog breeds and their close genetic relationship to wolves [31], but it could also point to the lack of proper socialization and training. Obedience training is often recommended for preventing and eliminating aggression in dogs [39].

Compulsive and aggressive behaviors in dogs can also be genetically conditioned [40,41,42,43,44]. Aggression has been linked with sex, origin and relationship with owners [39,45,46,47,48]. In this study, females tended to be more aggressive than males, whereas Borchelt [36] and Wright and Nesselrote [7] reported higher levels of aggression in males than in females.

The influence of a dog’s previous housing conditions on aggressive behavior was not confirmed in this study. However, present housing conditions were linked with aggressive behavior. The incidence of aggression was highest in dogs kept outdoors without confinement and in dogs kept indoors with backyard access. These behaviors indicate territorial aggression, which is a natural canine instinct resulting from the need to guard own territory against other dogs and animals [49]. Dogs naturally protect their territory where they feel safe and where their basic needs are met. Some owners unwittingly encourage territorial aggression, thus making their dogs overconfident and possessive of their territory [50]. These dogs are more likely to display territorial behaviors and act aggressively towards other animals. Beerda et al. [51] demonstrated that dogs kept in confined spaces with a limited number of environmental stimuli, including limited contact with humans, were more aggressive, excitable and anxious.

In this study, aggressive behaviors accounted for 41.41% of all undesirable behaviors (803 cases of aggression in a total of 1939 undesirable behaviors), including aggression towards other dogs/animals (33.47%, 649 cases) and humans (7.94%, 154 cases). These problems were most frequently encountered in dogs living indoors (in houses) with backyard access (120 cases of aggression) and dogs living indoors (in apartments) without backyard access (114 cases). Such behaviors are indicative of defensive aggression, which is fueled by fear and anxiety [50]. Dogs kept on a short leash do not feel confident, and the leash prevents the dog from defending itself against a perceived aggressor [50,52]. Dogs kept on a tight leashes probably also respond to the caretaker’s emotions [53]. In some cases, aggressive behavior is an expression of the dog’s innate need to defend the owner.

Other aggressive behaviors displayed during off-leash walking, playing or eating can be classified as competitive aggression [50,52]. In turn, aggression directed towards humans during play could be a sign of possessive aggression [50,52]. Dogs are strongly motivated to guard an important resource, such as a toy. Possession of an important resource asserts the dog’s status in the pack. Owners who enable such behaviors promote possessive instincts, and they risk being bitten when attempting to take away the toy [50,52].

The surveyed respondents also reported aggressive behaviors during grooming, which could be attributed to the fact that dogs were not familiar with the grooming routine or that grooming was painful. Ancient dog breeds have a thick hair coat, which needs to be regularly groomed [32]. Aggressive behaviors during grooming could result from irritation or fear [50]. A groomer invades a dog’s personal space; the dog is often immobilized and is unable to escape; therefore, aggression towards the groomer is the only mode of defense.

Separation anxiety is increasingly reported in dogs [54,55,56,57,58]. A survey involving American dog owners revealed that 17% of dogs that remain under veterinary care display clinical symptoms of separation anxiety [57]. A dog experiences acute stress when the owner is absent, which intensifies anxious behavior [58,59]. Separation anxiety is most often manifested by vocalization, destructive behavior, elimination in the house, salivation, licking and self-harm [60]. Behavioral symptoms also include pacing, spinning and other repetitive behaviors. However, vocalization, elimination and destruction of household items are sufficient to diagnose separation anxiety.

In this study, separation anxiety was not significantly influenced by breed, sex, origin or housing conditions. However, symptoms of separation anxiety were somewhat more prevalent in Alaskan Malamutes and Samoyeds than in Siberian huskies and Akitas.

The destructive behaviors reported in dogs that were left alone at home are indicative of separation anxiety. The most common undesirable behaviors were excessive vocalization, damage to furniture and other household objects, and scratching doors and windows. Alaskan Malamute was the most vocalizing breed, whereas Basenjis, Samoyeds and Siberian huskies engaged mostly in destructive behavior. These undesirable behaviors are indicative not only of separation anxiety but also of boredom. Dogs whose individual needs are not met, animals left home alone for long periods, and dogs that are not physically (exercise, walking, play) and mentally stimulated may display undesirable behaviors indicative of separation anxiety [4,61].

Most dogs vocalize in response to external stimuli, when guarding their territory, during interactions with other dogs and during play [62]. However, excessive and uncontrolled barking, whimpering and growling at night or during daytime can be classified as undesirable behavior. Excessive vocalization could be caused by medical, cognitive, psychological and behavioral problems [63].

In this study, Samoyeds were the most vocal breed, which vocalized excessively towards visitors, during interactions with other dogs, in response to commands, and when left alone at home. Similar to Samoyeds, many Basenjis also vocalized excessively when left home alone. Basenjis do not bark [50], and vocalization in the owner’s absence could imply that this breed is more prone to separation anxiety. Excessive vocalization was also reported in dogs living indoors or in outdoor kennels. Limited living space can lead to frustration [28,30,50]. Dogs living indoors are exposed to unknown sounds that reach the interior through windows and doors. Dogs housed in kennels can also see and smell people, animals and vehicles. In these cases, excessive vocalization could be a symptom of territorial behavior [50].

In the present study, the following behaviors were classified as undesirable locomotion behaviors: chewing legs or other body parts, excessive licking, tail chasing, running along the fence line, chasing cars and bicycles, and other behaviors. According to many researchers, excessive self-grooming, such as licking and chewing body parts, is a compulsive behavior [10,13,51,64]. When dermatological problems and pain are ruled out, excessive self-grooming could be a sign of a psychobiological condition, and it is often accompanied by anxiety and psychomotor agitation [6,16]. Severe compulsions point to serious behavioral problems, and they could indicate that the dog’s welfare has been compromised [28]. The most common undesirable motor behaviors observed daily include fence running when people, animals or vehicles pass by. Such behaviors are a manifestation of a natural hunting instinct rather than motor stereotypies. Motor stereotypies have been reported in dogs living in shelters [27] and kennels [64]. Spinning and jumping are the only types of physical activity that are available to dogs in confinement. In the absence of physical and mental stimulation, dogs engage in such activities to combat stress and boredom [4,5]. In the current study, undesirable motor activities were also more prevalent in dogs living indoors (in apartments) and animals kept in kennels.

In the present study, the incidence of undesirable behaviors differed across the examined ancient dog breeds. Undesirable behaviors were less prevalent in Siberian huskies than in the remaining breeds. In Akitas, Alaskan Malamutes, Samoyeds and Siberian huskies, self-aggression and compulsive tail-chasing were more prevalent motor activities than fence running and chasing moving vehicles. Car and bicycle chasing as a symptom of hunting aggression were particularly prevalent in Basenjis, and it accounted for 46.15% of all undesirable motor behaviors reported in this breed.

Dogs guard food and food bowls to protect resources that play an important role in their lives. Animals that feel threatened are more inclined to protect important resources, such as food [50]. Dogs are more likely to aggressively protect their resources if they are punished or intimidated by their owners. Previous housing conditions could also play a role. Animals that were homeless for long periods and had to compete for food with rivals are more likely to protect their resources in an aggressive manner [50].

The study confirmed that breed and present housing conditions affected undesirable behaviors in dogs. Aggression at mealtime was most prevalent in Alaskan Malamutes, followed by Siberian huskies, Akitas, Basenji and Samoyeds. An analysis of present housing conditions revealed that dogs living indoors with backyard access and dogs kept outside without confinement were most likely to develop undesirable feeding behaviors.

## 5. Conclusions

In the present study, breed, sex and housing conditions were linked with undesirable behaviors in ancient dog breeds. The breed was associated with aggression towards other dogs/animals, aggression towards humans, undesirable oral and locomotion behaviors, and excessive vocalization. Sex was linked only with aggressive behavior towards other dogs/animals, and aggressive behaviors were more prevalent in females than in males. Housing conditions were associated with aggression towards other dogs/animals, aggression at mealtime, and excessive vocalization.

Undesirable behaviors were most prevalent in Akitas, Siberian huskies and Samoyeds, and they were more frequent in males than in females. The incidence of undesirable behaviors was highest in dogs living indoors with and without backyard access. The most frequently reported behavioral problems in ancient dog breeds were aggression towards other dogs and animals, excessive vocalization, and undesirable motor activities.

## Figures and Tables

**Table 1:** Undesirable behaviors analyzed in the study.

Undesirable Behavior (Yes/No)	Description of Undesirable Behavior (the Respondents Could Select Multiple Answers)
Aggression towards other dogs/animals	- When walking on a leash - When walking off-leash - When eating - When playing - On own territory - Other
Aggression towards humans	- When playing - When eating - When being groomed - Towards visitors - On own territory - Other
Aggression at mealtime	- Guards food bowl - Guards food - Other
Separation anxiety	- Vocalizes - Eliminates in the house - Damages furniture and other objects - Scratches doors and windows - Other
Oral and locomotion behaviors	- Chews leg or other body parts - Self-licks excessively - Chases own tail - Runs along the fence line - Chases cars/bicycles - Other
Excessive vocalization	- When home alone - When meeting another dog during a walk - When responding to a command - Towards visitors - When guarding own terrain- Other

**Table 2:** The influence of breed on the incidence of undesirable behaviors in ancient dog breeds.

Behavior Problem		Akita (n = 253)		Alaskan Malamute (n = 116)		Basenji (n = 64)		Samoyed (n = 218)		Siberian Husky (n = 246)		χ^2^	*p*
		Yes	No	Yes	No	Yes	No	Yes	No	Yes	No		
Aggression towards other dogs/animals	n	149	104	54	62	35	29	25	193	86	160	122.739	0.001
	%	58.89	41.11	46.55	53.45	54.69	45.31	11.47	88.53	34.96	65.04		
Aggression towards humans	n	33	220	20	96	6	58	12	206	25	221	12.997	0.011
	%	13.04	86.96	17.24	82.76	9.38	90.63	5.50	94.50	10.16	89.84		
Aggression at mealtime	n	43	210	34	82	10	54	20	198	50	196	23.231	0.001
	%	17.00	83.00	29.31	70.69	15.63	84.38	9.17	90.83	20.33	79.67		
Separation anxiety	n	25	228	18	98	11	53	39	179	32	214	7.292	0.121
	%	9.88	90.12	15.52	84.48	17.19	82.81	17.89	82.11	13.01	86.99		
Oral and locomotion behaviors	n	69	184	27	89	14	50	64	154	44	202	10.018	0.040
	%	27.27	72.73	23.28	76.72	21.88	78.13	29.36	70.64	17.89	82.11		
Excessive vocalization	n	44	209	31	85	15	49	96	122	60	186	44.699	0.001
	%	17.39	82.61	26.72	73.28	23.44	76.56	44.04	55.96	24.39	75.61		

The results of the questionnaire survey were analyzed using Pearson’s chi-squared test (χ^2^). The results were regarded as significant at *p* ≤ 0.05.

**Table 3:** The influence of sex on the incidence of undesirable behaviors in ancient dog breeds.

Behavior Problem		Males (n= 460)		Females (n = 437)		χ^2^	*p*
		Yes	No	Yes	No		
Aggression towards other dogs/animals	n	195	264	154	283	4.821	0.028
	%	57.61	42.39	64.76	35.24		
Aggression towards humans	n	56	404	40	397	2.140	0.144
	%	12.17	87.83	9.15	90.85		
Aggression at mealtime	n	76	384	81	356	0.629	0.428
	%	16.52	83.48	18.54	81.46		
Separation anxiety	n	59	401	66	371	0.969	0.325
	%	12.83	87.17	15.10	84.90		
Oral and locomotion behaviors	n	123	337	95	342	3.045	0.081
	%	26.74	73.26	21.74	78.26		
Excessive vocalization	n	126	334	120	317	0.0005	0.982
	%	27.39	72.61	27.46	72.54		

The results of the questionnaire survey were analyzed using Pearson’s chi-squared test (χ^2^). The results were regarded as significant at *p* ≤ 0.05.

**Table 4:** The influence of origin on the incidence of undesirable behaviors in ancient dog breeds.

Behavior Problem		Kennel Registered with the Polish Kennel Club (n = 582)		Private Owner (n = 21)		Unregistered Kennel (n = 111)		Foundation (n = 92)		Shelter (n = 74)		Origin Unknown (n = 17)		χ^2^	*p*
		Yes	No	Yes	No	Yes	No	Yes	No	Yes	No	Yes	No		
Aggression towards other dogs/animals	n	215	367	12	9	51	60	38	54	28	46	5	12	7.100	0.213
	%	36.94	63.06	57.14	42.86	45.95	54.05	41.30	58.70	37.84	62.16	29.41	70.59		
Aggression towards humans	n	52	530	4	17	17	94	12	80	10	64	1	16	7.457	0.189
	%	8.93	91.07	19.05	80.95	15.32	84.68	13.04	86.96	13.51	86.49	5.88	94.12		
Aggression at mealtime	n	92	490	6	15	26	85	15	77	16	58	2	15	6.983	0.222
	%	15.81	84.19	28.57	71.43	23.42	76.58	16.30	83.70	21.62	78.38	11.76	88.24		
Separation anxiety	n	82	500	5	16	17	94	6	86	11	63	4	13	7.469	0.188
	%	14.09	85.91	23.81	76.19	15.32	84.68	6.52	93.48	14.86	85.14	23.53	76.47		
Oral and locomotion behaviors	n	157	425	4	17	20	91	15	77	20	54	2	15	9.910	0.078
	%	26.98	73.02	19.05	80.95	18.02	81.98	16.30	83.70	27.03	72.97	11.76	88.24		
Excessive vocalization	n	164	418	5	16	29	82	26	66	17	57	5	12	1.201	0.945
	%	28.18	71.82	23.81	76.19	26.13	73.87	28.26	71.74	22.97	77.03	29.41	70.59		

The results of the questionnaire survey were analyzed using Pearson’s chi-squared test (χ^2^). The results were regarded as significant at *p* ≤ 0.05.

**Table 5:** The influence of previous housing conditions on the incidence of undesirable behaviors in ancient dog breeds.

Behavior Problem		Indoors without Backyard Access (Apartment) (n = 147)		Indoors with Backyard Access (House) (n = 487)		Outdoors in a Kennel (n = 120)		Outdoors without Confinement (n = 69)		Outdoors with Confinement (n = 33)		Other (n = 41)		χ^2^	*p*
		Yes	No	Yes	No	Yes	No	Yes	No	Yes	No	Yes	No		
Aggression towards other dogs/animals	n	52	95	187	300	48	72	32	37	15	18	15	26	3.193	0.670
	%	35.37	34.63	38.40	61.60	40.00	60.00	46.38	53.62	45.45	54.55	36.59	63.41		
Aggression towards humans	n	11	136	51	436	12	108	11	58	4	29	7	34	5.476	0.361
	%	7.48	92.52	10.47	89.53	10.00	90.00	15.94	84.06	12.12	87.88	17.07	82.93		
Aggression at mealtime	n	27	120	86	401	24	96	11	58	4	29	5	36	2.181	0.824
	%	18.37	81.63	17.66	82.34	20.00	80.00	15.94	84.06	12.12	87.88	12.20	87.80		
Separation anxiety	n	24	123	64	423	13	107	14	55	6	27	4	37	5.336	0.376
	%	16.33	83.67	13.14	86.86	10.83	89.17	20.29	79.71	18.18	81.82	9.76	90.24		
Oral and locomotion behaviors	n	30	117	122	365	31	89	21	48	5	28	9	32	4.549	0.474
	%	20.41	79.59	25.05	74.95	25.83	74.17	30.43	69.57	15.15	84.85	21.95	78.05		
Excessive vocalization	n	38	109	141	346	27	93	18	51	10	23	12	29	2.486	0.779
	%	25.85	74.15	28.95	71.05	22.50	77.50	26.09	73.91	30.30	69.70	29.27	70.73		

The results of the questionnaire survey were analyzed using Pearson’s chi-squared test (χ^2^). The results were regarded as significant at *p* ≤ 0.05.

**Table 6:** The influence of present housing conditions on the incidence of undesirable behaviors in ancient dog breeds.

Behavior Problem		Indoors without Backyard Access (Apartment) (n = 422)		Indoors with Backyard Access (House) (n = 422)		Outdoors in a Kennel (n = 14)		Outdoors without Confinement (n = 39)		χ^2^	*p*
		Yes	No	Yes	No	Yes	No	Yes	No		
Aggression towards other dogs/animals	n	143	279	183	239	2	12	21	18	15.236	0.002
	%	33.89	66.11	43.36	56.64	14.29	85.71	53.85	46.15		
Aggression towards humans	n	38	384	52	370	1	13	5	34	2.800	0.424
	%	9.00	91.00	12.32	87.68	7.14	92.86	12.82	87.18		
Aggression at mealtime	n	58	364	90	332	2	12	7	32	8.509	0.037
	%	13.74	86.26	21.33	78.67	14.29	85.71	17.95	82.05		
Separation anxiety	n	61	361	51	371	4	10	9	30	6.517	0.089
	%	14.45	85.55	12.09	87.91	28.57	71.73	23.08	76.92		
Oral and locomotion behaviors	n	108	314	98	324	4	10	8	31	1.092	0.779
	%	25.59	74.41	23.22	76.78	28.57	71.43	20.51	79.49		
Excessive vocalization	n	135	287	96	326	5	9	10	29	9.601	0.022
	%	31.71	68.01	22.75	77.25	35.71	64.29	25.64	74.36		

The results of the questionnaire survey were analyzed using Pearson’s chi-squared test (χ^2^). The results were regarded as significant at *p* ≤ 0.05.

## Data Availability

Not applicable.

## Supplementary Materials

The following are available online at https://www.mdpi.com/article/10.3390/ani11051435/s1. Material S1: Questionnaire “Behaviors of dogs of selected ancient breeds”.
