# Supplementary material for: The Influence of Breed, Sex, Origin and Housing Conditions on Undesirable Behaviors in Ancient Dog Breeds
Source: Animals (Basel). 2021 May 17;11(5):1435. doi: 10.3390/ani11051435 (PMC8156398; doi:10.3390/ani11051435)
Supplement: Supplementary file 1 [file animals-11-01435-s001.zip › animals-1219662-supplementary.pdf]

Material S1. Questionnaire "Behaviors of dogs of selected ancient breeds"

Notes for respondents:
a/ Please complete the questionnaire only if you own a pedigree dog
b/ If you own more than one dog, complete the questionnaire separately for each dog

* Answer required

1. Dog's breed*
Choose only one:
- Akita
- Alaskan Malamute
- Basenji
- Samoyed
- Siberian Husky

2. Dog's sex*
- female
- male

3. Dog's origin*
- kennel registered with the Polish Kennel Club
- private owner
- unregistered kennel
- foundation
- shelter
- origin unknown

4. Housing conditions* (conditions in which the dog was kept as a puppy or the last known housing conditions)
- indoors without backyard access (apartment)
- indoors with backyard access (house)
- outdoors in a kennel
- outdoors without confinement
- outdoors with confinement
- other

5. Present housing conditions*
- indoors without backyard access (apartment)
- indoors with backyard access (house)
- outdoors in a kennel
- outdoors without confinement
- outdoors with confinement
- other

6. Is your dog aggressive towards other dogs/animals? *
- Yes
- No

7. If you selected "yes" in question 6, describe the situations in which your dog is aggressive towards other dogs/animals (skip this question if you selected "no" in question 6).

Select all answers that apply.

- when walking on a leash
- when walking off-leash
- when eating
- when playing
- on own territory
- other

8. Is your dog aggressive towards humans? *

- Yes
- No

9. If you selected “yes” in question 8, describe the situations in which your dog is aggressive towards humans (skip this question if you selected “no” in question 8)**.

- when playing
- when eating
- when being groomed
- toward visitors
- on own territory
- other

**Aggression towards visitors involves situations in which visitors are present in the same location as the dog. Aggression on the dog’s territory denotes aggressive behavior towards people that are known to the dog (such as the owner or the family) as well as passers-by and persons who approach the dog’s territory. The dog’s territory is defined as the backyard, apartment, outdoor kennel or dog bed.

10. Does your dog display aggressive behavior at meal time?*

- Yes
- No

11. If you selected “yes” in question 10, describe the type of aggressive behavior (skip this question if you selected “no” in question 10).

- guards food bowl
- guards food
- other

12. Is your dog accustomed to being left alone at home? *

- Yes
- No

13. If you selected “no” in question 12, describe the undesirable behaviors your dog engages in when left alone at home(skip this question if you selected “no” in question 12).

- vocalizes
- eliminates in the house
- damages furniture and other objects
- scratches doors and windows
- other

14. Does your display undesirable oral and locomotion behaviors? *

- Yes
- No

15. If you selected “yes” in question 14, describe the type of undesirable oral and locomotion behaviors (skip this question if you selected “no” in question 14).

- chews leg or other body parts
- self-licks excessively
- chases own tail
- runs along the fence line
- chases cars/bicycles
- other

16. Does your dog vocalize excessively? *

- Yes
- No

17. If you selected “yes” in question 16, describe the situations in which your dog vocalizes excessively (skip this question if you selected “no” in question 16).

- when home alone
- when meeting another dog during a walk
- when responding to a command
- toward visitors
- when guarding own terrain
- other
